# Cultural and Digital Health Literacy Appropriateness of App- and Web-Based Systems Designed for Pregnant Women With Gestational Diabetes Mellitus: Scoping Review

**DOI:** 10.2196/37844

**Published:** 2022-10-14

**Authors:** Yosefa Birati, Enav Yefet, Yuri Perlitz, Naim Shehadeh, Sivan Spitzer

**Affiliations:** 1 The Azrieli Faculty of Medicine Bar-Ilan University Safed Israel; 2 Department of Obstetrics and Gynecology Baruch Padeh Medical Center Poriya Tiberias Israel; 3 Institute of Endocrinology, Diabetes and Metabolism Rambam Health Care Campus Haifa Israel

**Keywords:** gestational diabetes mellitus, maternal health, mobile health, mHealth, mobile apps, mobile phone, telemedicine, culture, health literacy, vulnerable populations, pregnancy outcome

## Abstract

**Background:**

The prevalence of women diagnosed with gestational diabetes mellitus (GDM) is increasing dramatically. Mobile technologies to enhance patient self-management offer many advantages for women diagnosed with GDM. However, to our knowledge, although mobile health (mHealth) and telemedicine systems for GDM management exist, evidence on their cultural and digital health literacy appropriateness levels is limited.

**Objective:**

This review aimed to search and assess the literature on mHealth and telemedicine systems designed for women diagnosed with GDM. Our assessment of these technologies focused on their cultural and digital health literacy appropriateness as well as the systems’ effectiveness in improving glycemic control and maternal and infant outcomes.

**Methods:**

We conducted a scoping review using a framework adapted from Arksey and O’Malley. Four electronic databases were searched for relevant studies: PubMed, MEDLINE (EBSCO), Web of Science, and Scopus. The databases were searched between January 2010 and January 2022. The inclusion criteria were pregnant women diagnosed with GDM, use of telemedicine for monitoring and management, and vulnerable or disadvantaged patients. We used terms related to mobile apps and telemedicine: *GDM*, *vulnerable populations*, *periphery*, *cultural appropriateness*, and *digital health literacy*. Studies were screened and selected independently by 2 authors. We extracted the study data on a Microsoft Excel charting table and categorized them into final themes. The results were categorized according to the cultural and digital health literacy features presented.

**Results:**

We identified 17 studies that reported on 12 telemedicine and mHealth app interventions. We assessed the studies in three domains: cultural appropriateness, digital health literacy, and maternal and infant outcomes. In the literature, we found that existing digital technologies may improve glycemic control and diabetes self-management. However, there is a lack of assessment of cultural and digital health literacy appropriateness for pregnant women diagnosed with GDM. Considerations in app design regarding cultural appropriateness were found in only 12% (2/17) of the studies, and only 25% (3/12) of the interventions scored ≥3 out of 5 in our assessment of digital health literacy.

**Conclusions:**

mHealth and telemedicine can be an effective platform to improve the clinical management of women with GDM. Although studies published on the use of mHealth and telemedicine systems exist, there is a limited body of knowledge on the digital health literacy and cultural appropriateness of the systems designed for women diagnosed with GDM. In addition, as our study was restricted to the English language, relevant studies may have been excluded. Further research is needed to evaluate, design, and implement better tailored apps regarding cultural and digital literacy appropriateness for enhancing pregnant women’s self-management as well as the effectiveness of these apps in improving maternal and infant health outcomes.

## Introduction

### Background

The prevalence of women diagnosed with diabetes during pregnancy has substantially increased over the last decade. The International Diabetes Federation 2019 report estimated that 20 million women developed hyperglycemia during pregnancy, 84% of them because of gestational diabetes mellitus (GDM) [1]. GDM is associated with significant risk and can lead to grave adverse perinatal outcomes and long-term health complications for both mothers and their offspring [2-4]. Self-care and changes in lifestyle are essential for adequate glycemic control and prevention of unfavorable maternal-infant health outcomes [5,6]. GDM management requires women to implement medical nutrition therapy, self-monitor their blood glucose levels, manage weight gain, and perform physical exercise [7-9]. These self-management tasks are complex and pose a significant self-care burden for pregnant women, especially those who are diagnosed for the first time.

Digital technology solutions have been introduced to support and improve women’s management and outcomes while decreasing the need for direct physician-patient contact. Digital health platforms include mobile health (mHealth) apps, telehealth, and telemedicine, and the information can be delivered through a wide range of technologies such as web-based services, mobile devices, and software systems. These platforms are perceived as a way to reduce disparities in access and quality of care for patients living in rural areas [10]. However, although these technologies have many potential advantages, the extent to which they address the needs of women with diverse communication competencies, culture, and language and different health and digital literacy levels remains unclear [11,12].

Digital health literacy is defined as “the ability to seek, find, understand, and appraise health information from electronic sources and apply the knowledge gained to address or solve a health problem” [13]. As both health care systems and providers gradually increase their use of health technologies, patients are asked in turn to engage with advanced digitalization, posing additional barriers. Recent studies have shown that low health literacy is positively correlated with deficiencies in diabetes knowledge and self-management among patients with diabetes [14-16] and an increase in health care provider workload [17]. In 2013, the Institute of Medicine published a discussion paper suggesting strategies for improving health literacy and usability by developing health literate apps [18]. However, evidence on the use of mHealth apps and telemedicine systems is based mainly on studies conducted on the general population. Thus, it is not clear if and how levels of digital and health literacy were considered in the development of these systems [19]. Moreover, the Academy of Nutrition and Dietetics guidelines for GDM (2018) suggested that sociocultural assessments such as religious dietary restrictions, food insecurity, or fasting related to religious beliefs should be addressed according to the patient’s needs because of their influence on pregnant women’s lifestyle and self-management [20]. Cultural appropriateness and cultural sensitivity assessments are essential in the design of digital mHealth apps and telemedicine systems developed for improved GDM management.

### Objectives

To our knowledge, reviews assessing digital health literacy and cultural appropriateness of mHealth and telemedicine systems developed for women with GDM have not been conducted. Given the limited evidence, the main objective of this review was to search the literature on mHealth and telemedicine systems designed for women with GDM and assess their cultural and digital health literacy appropriateness as well as the systems’ effectiveness in improving glycemic control and maternal and infant outcomes.

## Methods

### Overview

We conducted this scoping review following the methodological framework proposed by Arksey and O’Malley [21] and Levac et al [22] considering the further refinements made by the Joanna Briggs Institute Reviewers’ Manual [23]. The framework by Arksey and O’Malley is based on six essential stages: (1) identifying the research question; (2) searching and identifying relevant studies; (3) selecting the relevant studies; (4) charting the data; (5) collating, summarizing, and reporting the results; and (6) consulting with stakeholders (optional). We selected the scoping review methodology as our aim was to explore the current body of knowledge regarding GDM mHealth apps tailored for cultural and digital health literacy appropriateness, identify existing knowledge and implementation gaps, and suggest future research needed. Furthermore, the reporting of this scoping review was guided by the PRISMA (Preferred Reporting Items for Systematic Reviews and Meta-Analyses) extension for Scoping Reviews (PRISMA-ScR) checklist [24] (Multimedia Appendix 1).

### Search Strategy

Four electronic databases—PubMed, MEDLINE (EBSCO), Web of Science, and Scopus—were searched using the following terms: (1) *telemedicine*, (2) *gestational diabetes mellitus*, (3) *target vulnerable populations*, (4) *remote/periphery areas*, (5) *culture appropriate*, and (6) *digital health literacy*. An example of the search strategy and keyword combination for the PubMed database can be found in Multimedia Appendix 2.

### Inclusion and Exclusion Criteria

Studies were included based on the inclusion and exclusion criteria and if they met the participants or population, concept, and context mnemonic categorization recommended by the Joanna Briggs Institute for scoping reviews (Table 1).

In our initial search, we examined studies published between January 2010 and October 2021. We updated our search to ensure that any new relevant publications between October 2021 and January 2022 were included. Protocols and feasibility studies for mHealth and web-based system design and development were not included, but they informed our search for publications that presented implementation and study outcomes. Similarly, relevant review publications were not included, but their reference lists were hand searched for additional original papers potentially eligible for inclusion in this scoping review. In addition, we scanned the reference lists of all studies selected for inclusion for additional relevant studies.

**Table 1:** Inclusion and exclusion criteria.

Categorization	Inclusion criteria	Exclusion criteria
Participants or population	Pregnant women who were diagnosed with GDM^a^	Nonpregnant patients Pregnant women who were not diagnosed with GDM
Concept	Use of mHealth^b^ for GDM monitoring and management mHealth was considered as telemedicine, mobile phone apps, smartphone apps, and web-based systems	Use of mHealth telemonitoring for patients who were not diagnosed with GDM (eg, postpartum follow-up, pregnant patients who were diagnosed with type 1 or type 2 diabetes, patients with diabetes who were not pregnant, use of mHealth for pregnant patients following HTN^c^, and fetal monitoring)
Context	Vulnerable or disadvantaged patients or groups (ethnic minorities, migrants, underserved populations, and digital health literacy) Rural and underserved areas and periphery	N/A^d^
Type of studies	Qualitative, quantitative, or mixed methods studies Observational and experimental, cross-sectional, or longitudinal; RCT^e^, nonrandomized, or noncontrolled trials; and case series or case reports	Conference abstracts, editorials, commentaries, letters to editor, essays, book chapters, and books
Language	English	Languages other than English

^a^GDM: gestational diabetes mellitus.

^b^mHealth: mobile health.

^c^HTN: hypertension.

^d^N/A: not applicable.

^e^RCT: randomized controlled trial.

### Screening and Selection of Studies

**Figure 1:**
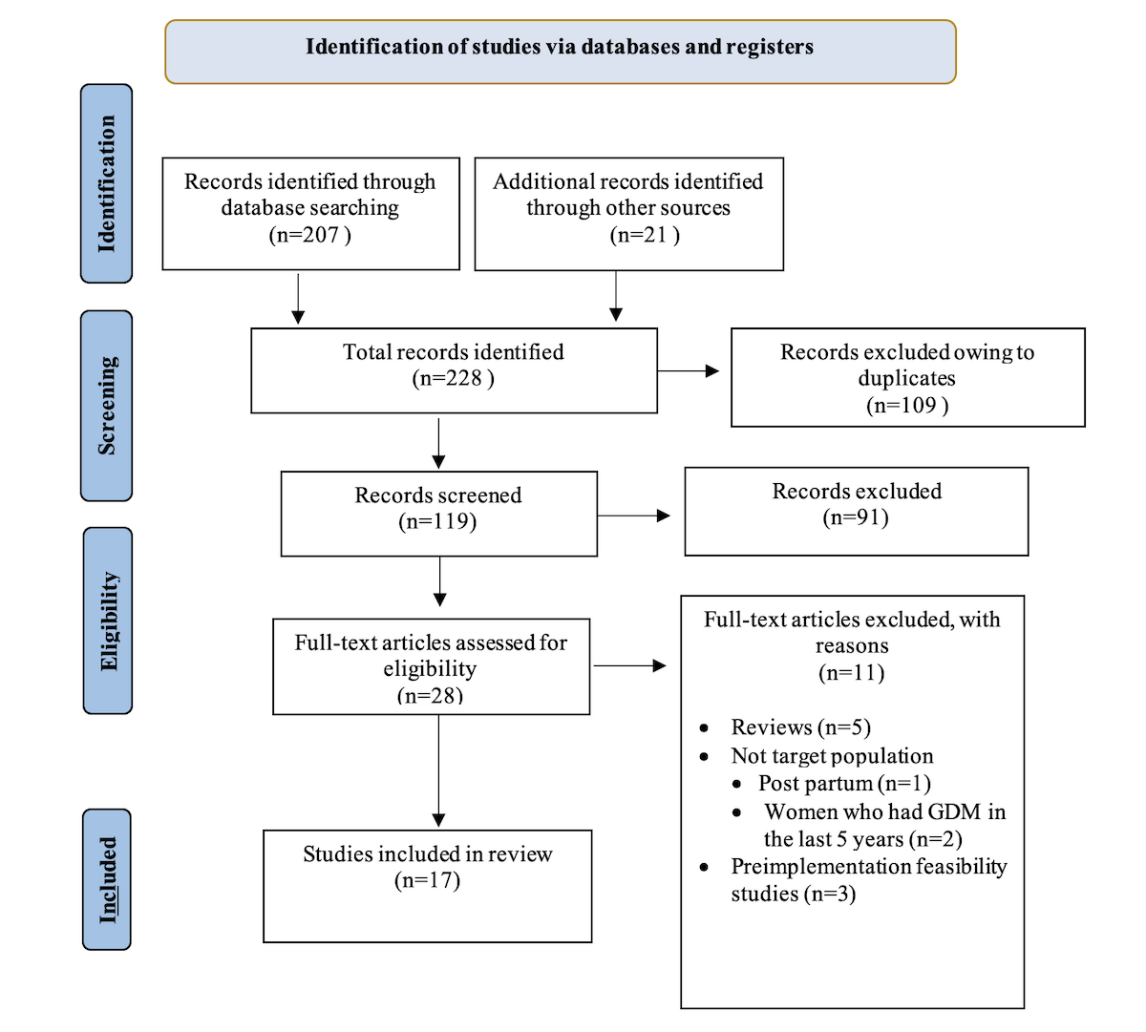
PRISMA (Preferred Reporting Items for Systematic Reviews and Meta-Analyses) flowchart.

Our initial search of 4 databases yielded 207 results. Our hand search identified 21 additional records. After duplicates were removed, 52.2% (119/228) of publications were reviewed. The selection procedure is presented in the PRISMA flow diagram (Figure 1). The titles and abstracts of 52.2% (119/228) of the articles were screened independently by 2 authors (YB and SS). Following initial screening, of the 119 articles, 91 (76.5%) were excluded, including 5 (4.2%) reviews whose reference lists were searched. The remaining 28 publications’ full texts were reviewed and screened for eligibility. A total of 11 publications were excluded (reviews: n=5, 45%; not the target population and women who had GDM but the study was conducted during their postpartum period: n=1, 9%; nonpregnant women who were diagnosed with GDM in the last 5 years: n=2, 18%; and preimplementation usability and feasibility studies: n=3, 27%). Disagreements in the decisions were resolved through discussion and consensus.

### Charting the Data

The information we derived from the studies included in this scoping review was recorded using a Microsoft Excel (Microsoft Corp) data charting table. The table included general information on the study characteristics (title, year of publication, country, research aims, study design, and population), description of telemonitoring intervention versus usual standard of care, evaluation of digital health literacy, cultural features, outcome measures, and results.

### Collating, Summarizing, and Reporting the Results

Selected papers were evaluated thoroughly by both reviewers to identify similarities and differences in mHealth interventions and were summarized for telemedicine and app development and culturally appropriate design. We also evaluated the degree to which these apps addressed health literacy by assessing five digital health literacy features: (1) patients’ ability to use a smartphone for chatting, reading, and writing; (2) patient training and guidance on how to use the technology; (3) plain language; (4) display and organization of information (simplified navigation); and (5) the intervention being tested for feasibility and usability. An additional search was conducted to better assess the feasibility and usability of the apps of the interventions included in this scoping review. We searched for previous studies conducted by the authors to evaluate their interventions’ feasibility before the final implementation. The outcome measures and significant results were summarized. The data were categorized and organized into final themes.

## Results

A total of 17 articles were included for data extraction in this scoping review, as can be seen in the PRISMA flowchart (Figure 1).

### Characteristics of the Studies

The included studies were published between 2010 and 2021. Of the 17 articles, 4 (24%) were published in Norway [25-28], and 3 (18%) were published in Spain [29-31], followed by China (n=3, 18%) [32-34], Singapore (n=2, 12%) [35,36], the United States (n=1, 6%) [37], Australia (n=1, 6%) [38], France (n=1, 6%) [39], the United Kingdom (n=1, 6%) [40], and Israel (n=1, 6%) [41]. A total of 35% (6/17) of the studies were multicenter randomized controlled trials (RCTs) [25,26,32,33,37,38], 29% (5/17) were single-center RCTs [29,34,36,40,41], 6% (1/17) were non-RCTs [30], 6% (1/17) had an experimental design [31], 18% (3/17) were qualitative studies [27,28,39], and 6% (1/17) had a mixed methods design [35]. The study interventions included a web-based telemedicine system [30-33,37-39], web-based applications [29,35,36,41], and mHealth apps [25-28,34,40] (Table 2).

**Table 2:** Characteristics and cultural appropriateness of the 12 apps and systems.

Study	Study design	Country	Study population	Race and ethnicity	Intervention	Cultural appropriateness
Tian et al [32], 2021	RCT^a^	China	GDM^b^ IG^c^: n=133 CG^d^: n=136	Ethnic Han: 96.3% Other: 3.7%	WeChat	+^e^ Individualized guidance for self-management
Yang et al [33], 2018	RCT	China	GDM IG: n=57 CG: n=50 Normal glucose tolerance: n=50	N/A^f^	WeChat	+/−^g^ Individualized dietary advice
Homko et al [37], 2012	RCT	United States	GDM IG: n=36 CG: n=38	African American: 30% White: 37.5% Latino or Hispanic: 20% Asian and other: 12.5%	Web-based system	−^h^
Garnweidner-Holme et al [26], 2020	RCT	Norway	GDM IG: n=95 CG: n=98	Norway: 46.6% Western Europe+United States: 6.7% Eastern Europe: 9.3% Asia: 23.3% Africa: 11.4% South America: 2.6%	Pregnant+ app	+ Norwegian, Urdu, or Somali language and food culture
Borgen et al [25], 2019	RCT	Norway	GDM IG: n=115 CG: n=123	Norway: 46.8% Western Europe+United States: 5.9% Eastern Europe: 8.9% Asia: 23.6% Africa: 12.7% South America: 2.1%	Pregnant+ app	+ Norwegian, Urdu, or Somali language
Albert et al [31], 2020	Descriptive—clinical trial	Spain	GDM N=20	N/A	Web-based system SineDie	−
Pérez-Ferre et al [29], 2010	RCT	Spain	GDM IG: n=48 CG: n=49	IG: White: 51% Hispanic: 30.6% Asian: 6.1% North African: 4.1% Other: 8.2% CG: White: 56.2% Hispanic: 37.5% Asian: 4.2% North African: 2.1%	Web internet-based application	−
Surendran et al [35], 2021	RCT and qualitative; mixed methods	Singapore	GDM Quantitative data: n=170 Semistructured interviews: n=14	Quantitative data Chinese: 44% Non-Chinese: 56% Interviews Chinese: 57% Non-Chinese: 43%	Web-based application Habits-GDM	− Limited common local and ethnic food database (64%) Food item’s name was not worded in the commonly known way, and the imperial measurement (cup) was not familiar to the participants
Carral et al [30], 2015	Prospective interventional study	Spain	Pregnant women with diabetes GDM: n=77 Type 1 DM^i^: n=16 Type 2 DM: n=11 IG: n=40 CG: n=64	White: 96.2% Hispanic: 1.9% North African: 1.9%	Web-based telemedicine platform DiabeTIC	−
Guo et al [34], 2019	RCT	China	GDM IG: n=64 CG: n=60	N/A	mHealth^j^ app Dnurse app	−/+ Personalized dietary guidance
Mackillop et al [40], 2018	RCT	United Kingdom	GDM IG: n=101 CG: n=102	IG: White: 77% South Asian: 10% African or Caribbean: 6% East Asian: 3% Other: 4% CG: White: 78,4% South Asian: 12.7% African or Caribbean: 3.9% East Asian: 1% Other: 3.9%	mHealth app	−
Miremberg et al [41], 2018	RCT	Israel	GDM IG: n=64 CG: n=60	N/A	Web-based application	−
Yew et al [36], 2021	RCT	Singapore	GDM IG: n=170 CG: n=170	IG: Chinese: 44.1% Non-Chinese: 55.9% CG: Chinese: 43.5% Non-Chinese: 56.5%	Web-based application Habits-GDM	−/+ A database of common foods in Singapore was incorporated into the app A manuscript written by Surendran et al [35] on the same study found that the food items’ names were not worded in the commonly known way, and the imperial measurement (cup) was not familiar to the participants
Skar et al [28], 2019	Qualitative	Norway	GDM N=17	Norway: 59% Immigrants (Poland, Bulgaria, Turkey, Pakistan, Palestine, and Sweden): 41%	N/A	+ Information about health and nutrition in Norwegian, Urdu, and Somali
Rasekaba et al [38], 2018	RCT	Australia	GDM IG: n=61 CG: n=34	N/A	Web-based telemedicine platform TeleGDM	−
Khalil [39], 2019	Qualitative	France	GDM n=5 Health care providers: Diabetes specialists (n=8) Educational nurses (n=8) Dietitians (n=2) Gynecologists (n=1) Midwives (n=1)	N/A	Telemonitoring system myDiabby	−

^a^RCT: randomized controlled trial.

^b^GDM: gestational diabetes mellitus.

^c^IG: intervention group.

^d^CG: control group.

^e^Considered the issue.

^f^N/A: not applicable.

^g^Partially considered the issue.

^h^Did not consider the issue.

^i^DM: diabetes mellitus.

^j^mHealth: mobile health.

### Culturally Appropriate Intervention Design

Cultural appropriateness is defined as “the ability to recognize, understand, and react appropriately to beliefs, values, norms, and behaviors of persons who belong to a cultural or ethnic group that differs from one’s own” [42]. We operationally defined culturally appropriate design as the assessment and awareness of the researchers in the intervention’s design phase in adapting the app’s content and instructions according to the patients’ culture, language, religion, customs, and beliefs. Assessment and design of cultural appropriateness was considered in only 17% (2/12) of the telemedicine and app interventions we identified. In the Pregnant+ app study, for example, the authors acknowledged the high prevalence of GDM among immigrant women in Norway and the importance of designing and incorporating linguistic and culturally adapted information. The app was translated into 3 languages and also included preferred food items according to culture [25-28,43]. In the Habits-GDM application design, researchers included a database of common foods in Singapore [36]. However, in a qualitative study that examined the Habits-GDM application users’ perceptions, 9 out of the 12 interviewed women stated that the database had limited ethnic foods, and 12 out of 14 women claimed that the measurement units were not familiar to them [35]. Another 12% (2/17) of the studies assumed and stated in their study limitations that their targeted users were users with high levels of cultural literacy, but their sample did not represent high-risk or low socioeconomic groups and, therefore, no cultural modifications were added [34,41]. Another 6% (1/17) of the studies acknowledged the existing gaps among rural and disadvantaged populations, which caused the women to avoid using the telemedicine system [30].

### Digital and Health Literacy

#### Overview

We evaluated the 12 intervention studies according to the 5 digital health literacy features, as presented in Table 3, providing an overall maximum score of 5 on the extent to which the apps addressed the 5 features of digital health literacy (Table 3).

**Table 3:** Summary of the included studies’ digital and health literacy features (N=14).

Study	Ability to use a smartphone for chatting, reading, and writing	Proper training and guidance	Content—plain language	Health content—displayed and organized (simplified navigation)	Test usability	Overall digital and health literacy score out of 5
Tian et al [32], 2021	−^a^ Inclusion criteria: ability to use a smartphone for chatting, reading, and writing basic Chinese	N/A^b^	N/A	N/A	−/+^c^ Brief interviews	0.5
Yang et al [33], 2018	− Study exclusion criteria: inability to operate a mobile phone or WeChat	+^d^ The research team taught the patients how to use the app	N/A	N/A	N/A	1
Homko et al [37], 2012	− A total of 7 patients (22%) in the IG^e^ never accessed the system	+ IG received training following installation. A total of 3 patients (20%) needed additional training or to correct technical problems	N/A	+	+ Test usability [44]	3
Garnweidner-Holme et al [26], 2020	+	− Relied on the women’s own capability to download and use the app	+ Information was in line with the varying levels of literacy The content writing, literacy, and visual communication were assessed against the Kreuter message checklist [45]	+ A multidisciplinary research team and experts in software were involved in the design and development and data privacy and security, as well as a graphic designer and language editor The content was ordered using 4 icons	+ A total of 21 pregnant women were involved in the development phase and gave feedback, and adjustments were made [43]	4
Borgen et al [25], 2019	+	−	+	+	+	4
Albert et al [31], 2020	N/A	N/A	N/A	+ Monitoring data were presented in an electronic logbook	+ Before insulation, the system was evaluated for validity, safety, and effectiveness [46]	2
Pérez-Ferre et al [29], 2010	− A total of 10 women were excluded because of inability to understand or comply with the protocol	− A total of 5 patients were not able to transmit any data No further intervention was described to enhance the training These patients had a lower educational level or difficulties with the language or were not used to new technologies	N/A	N/A	+ Feasibility test [47]	1
Surendran et al [35], 2021	+ Half of the app users (84/170) accessed at least one educational lesson	N/A	+ Content of the educational lessons was easy to understand (6/9 women)	− All information in one place (3/9 women); difficult to search features in the diet-tracking function (3/12 women)	−/+ A qualitative study of the patients’ experience was conducted after the study trial [36] No pilot study was conducted	2.5
Carral et al [30], 2015	+ Satisfaction survey—the platform is easy to use: mean 8.1 (SD 1.5, range 5-10)	N/A	N/A	+ Satisfaction survey—navigation through the platform is intuitive: mean 6.7 (SD 3.0, range 1-10); allows me to adequately visualize the information: mean 8.2 (SD 1.8, range 4-10)	+ A pilot study examined patient satisfaction [48]	3
Guo et al [34], 2019	− Inclusion criteria: patients with smartphones and proficiency in the use of mobile apps Most of the patients already had a high level of digital literacy	N/A	N/A	N/A	N/A	0
Mackillop et al [40], 2018	− Researchers assumed that the women enrolled in the study had high rates of literacy	N/A	N/A	+ Following the app development test, displays were changed to show BG^f^ readings in both graphical and tabular formats with color-coded thresholds [49] Illustrations were added to on-screen buttons	+ Testing the app development [49]	2
Miremberg et al [41], 2018	− Inclusion criteria: ability to speak English at least to a level that enabled the women to use the application and communicate with the clinic team (the study was conducted in Israel)	N/A	−	−/+ Patients reported “high” or “very high” satisfaction with their application-based prenatal care In total, 80% of the patients reported no difficulty using the application (20% of the patients reported slight difficulty mainly related to the English language barrier)	N/A	0.5
Yew et al [36], 2021	− Inclusion criteria: proficiency in English (the study was conducted in Singapore)	N/A	N/A	− Patients were required to be able to navigate an application	N/A	0
Rasekaba et al [38], 2018	+ Barriers: financial disadvantage in accessing the service and level of technological literacy [50]	N/A	N/A	N/A	+ Patient satisfaction survey and provider usability [51]	2

^a^Did not consider the issue.

^b^N/A: not applicable.

^c^Partially considered the issue.

^d^Considered the issue.

^e^IG: intervention group.

^f^BG: blood glucose.

#### Women’s Ability to Use a Smartphone for Chatting, Reading, and Writing

We evaluated whether the interventions assessed their patients’ levels of digital health literacy before the design and development of the intervention, such as women’s ability to use a smartphone for chatting, reading, and writing. The Pregnant+ app was the only one of the 12 interventions in which the content was checked during its app design and development phase using the Kreuter health message checklist with regard to patients’ ability to read the app information [45] and that also assessed the suitability of the materials [52]. Following this evaluation, researchers added explanations of diabetes medical terms to the app, and the women received information in accordance with their different literacy levels [43]. In DiabeTIC, a pilot study was conducted on a sample of patients with diabetes not exclusive to GDM to evaluate participants’ satisfaction with the telemedicine platform monitoring and metabolic control. A total of 7 out of 32 participants were women diagnosed with GDM. The overall mean score from study participants on the item “the platform is easy to use” was 8.1 (SD 1.5, range 5-10) [30,48]. Rasekaba et al [50] identified in their study that barriers to using the app were due to the women’s level of digital literacy and technology proficiency. However, this assessment was conducted after the start of the project [50]. In another 24% (4/17) of the studies, those who were not able to use a mobile phone or had language difficulties were excluded in the enrollment phase [32-34,36,41]. Of these 4 studies, 2 (50%) required proficiency in English even though, in 50% (1/2) of those studies, the country’s official language was not English and, in the other 50% (1/2), English was one of 4 official languages [36,41]. Mackillop et al [40,53] assumed that the women they enrolled in their study already had high rates of literacy and did not mention any assessment regarding digital literacy (Table 3).

#### Patient Training and Guidance on How to Use the Technology

A total of 18% (3/17) of the studies reported that they conducted training for the pregnant women who participated in the intervention group. In the WeChat intervention, the research team taught the women how to use the app [33]. Homko et al [37] acknowledged the existing digital challenges and educated the women on how to use the technology. In a previous feasibility test, 15 women (47%) received computers, internet access, and a training session. Three of these women needed additional training. However, 22% of the women in the intervention group did not access the telemedicine system or use it. In the study limitations, the authors reported that, out of an average of 8 weeks of follow-up, the women used and transmitted their measurements on an average of only 3 weeks [37,44]. In the Pregnant+ app, researchers assumed that the women were capable of downloading and using the app, and no training was offered [25]. In the rest of the studies (11/17, 65%), the information was not reported (Table 3).

#### Plain Language

The Pregnant+ app recognized the different literacy levels of the women. Thus, they amended the information that the women received in line with their needs. Here, too, contents were checked using the Kreuter health message checklist to assess developers’ use of plain language [43,45]. Regarding Habits-GDM, interviews were conducted after the study trial. A total of 6 out of 9 women (67%) said that the educational lessons were easy to understand [35]. In all other interventions, information regarding the level of language and medical jargon used was not available (Table 3).

#### Display and Organization—Simple Navigation

Navigation and screen display were described in 25% (3/12) of the interventions. In the Pregnant+ app, a graphic designer and language editor were involved in the design and development phases. The app content was designed and organized hierarchically and included only 4 icons to ease use and avoid overburdening the pregnant women [43]. Homko et al [44], in a preceding feasibility study, described the system’s web screens for measurements and information, the data entry section, the sent questions, and the data appearance. Regarding the SineDie application, a previous study was conducted to evaluate the system. In the manuscript, the authors described the system design and architecture and included photos to demonstrate the view and drop-down lists that the women used when entering data as well as the summary of the electronic logbook [31,46]. In the Habits-GDM enrollment phase, those who were included in the study were required to be able to use a mobile phone and navigate through the application. The intervention interviews conducted showed that only 3 out of 9 users (33%) reported that all the information was in one place, and 3 out of 12 users (25%) said that they had difficulties in searching for features in the diet-tracking function while using the application [35,36]. A satisfaction survey conducted on the DiabeTIC web-based telemedicine platform found that the women’s mean score on the item “understanding how to navigate through the platform” was 6.7 (SD 3.0, range 1-10), and the mean score on the item “adequate visualization of all information” was 8.2 (SD 1.8, range 4-10) [30,48]. Mackillop et al [49] tested the usability and reliability of the app and, following the results, app displays and colors were changed. In addition, to ease app navigation, they added illustrations on the screen icons [40,49] (Table 3).

#### Testing the Intervention

We found that 50% (6/12) of the interventions conducted preimplementation studies to examine user experience, ease of use, understanding of content, and app navigation following the prototype’s design and development. Homko et al [44] tested the intervention’s feasibility focusing on how well the pregnant women communicated with their health care provider and used the telemedicine system for better maternal and infant outcomes. The Pregnant+ app involved 21 pregnant women in its design and development phase, and 2 user-involvement studies were conducted afterward [43]. In addition, 2 qualitative studies were carried out to examine women’s and providers’ experiences and attitudes toward the Pregnant+ app [27,28]. Regarding the SineDie web-based clinical decision support system, a feasibility study was conducted. A total of 25 women participated in a validity study, and 90 women were randomized and participated in a clinical trial study testing effectiveness [46]. Mackillop et al [49], before the implementation of their intervention, conducted beta testing for system use (n=7) and the service development phase (n=48). Pérez-Ferre et al [47] conducted a pilot study to test the telemedicine system’s feasibility in clinical practice, reduction of face-to-face visits, and participants’ satisfaction. The DiabeTIC pilot study examined 32 participants’ satisfaction levels with the use of the telemedicine platform. A total of 7 of the 32 patients were women diagnosed with GDM [48]. In total, 25% (3/12) of the interventions evaluated the mHealth apps and telemedicine systems during implementation or afterward. In the WeChat intervention, short interviews were conducted, but no details on the questions and answers were described in the manuscript [32]. Regarding Habits-GDM, a qualitative study examined patients’ experiences using the app after the study trial was delivered [36]. The TeleGDM web-based telemedicine study used mixed methods to examine patient and provider usability, acceptance, and satisfaction with using the technology [51] (Table 3).

### Effectiveness

#### Overview

A summary of the outcome measurements and significant results of the 82% (14/17) of quantitative studies included in this review is provided in Table 4.

**Table 4:** Maternal and neonatal outcome measurements and significant results (N=14).

Study	Maternal clinical outcome measurements	Pregnant women’s lifestyle outcome measurements	Neonatal outcome measurements	Significant outcomes
Tian et al [32], 2021	Glycemic control: Number of BG^a^ levels within the control range FBG^b^ and 2hBG^c^ Maternal outcomes: PROM^d^ Postpartum hemorrhage Delivery mode—CS^e^, vaginal, or vacuum	N/A^f^	Preterm birth Birth weight	Differences in glycemic qualification, but clinical maternal and neonatal outcomes were not significantly different between the IG^g^ and CG^h^
Yang et al [33], 2018	Glycemic control: FBG 1hBG^i^ 2hBG Maternal outcomes: Delivery mode—CS, vaginal, or vacuum PIH^j^ PROM	N/A	Birth weight or macrosomia Admission to the NICU^k^ Neonatal jaundice or hyperbilirubinemia Neonatal hypoglycemia Preterm birth	Glycemic control: FBG: P<.001 (IG vs CG) 2hBG: P<.001 (IG vs CG) Maternal outcomes: Premature delivery (IG vs CG): P=.03 CS was more likely in the IG: P=.03
Homko et al [37], 2012	Glycemic control: FBG Maternal outcomes: Delivery mode—CS, vaginal, or vacuum PIH or pre-eclampsia PROM Gestational week of delivery	Use of the system	Admission to the NICU Birth weight or macrosomia Apgar score Neonatal hypoglycemia Respiratory morbidities	Women who used the internet sent more transmissions than women who used the phone or IVR^l^ system: P=.007 Women with higher incomes transmitted more frequently: P<.01
Garnweidner-Holme et al [26], 2020	Maternal outcomes: Dietary changes	N/A	N/A	No significant differences
Borgen et al [25], 2019	Glycemic control: 2-hour glucose level postpartum OGTT^m^ Maternal outcomes: Delivery mode—CS, vaginal, or vacuum	Engagement with health	Birth weight or macrosomia Admission to the NICU Apgar score	Women in the IG were less likely to have an emergency CS compared with the CG—overall mode of delivery: P=.03. However, when the women were stratified by parity, this difference was no longer statistically significant Higher number of women reported that apps made them more engaged with their health: P<.01 However, a single self-constructed, nonvalidated question was used to measure this, and it was not specific to the intervention app
Pérez-Ferre et al [29], 2010	Glycemic control: Change in HbA1c Maternal outcomes: PIH or pre-eclampsia Delivery mode—CS, vaginal, or vacuum Weight gain Gestational week of delivery BP^n^	Number of outpatient visits	Birth weight or macrosomia Admission to the NICU Neonatal hypoglycemia Preterm birth Shoulder dystocia	Reduction in outpatient clinic visits in women from the telemedicine group (P<.03) The women in the IG had more contacts with health personnel and took up less time (P<.001) than those in the CG
Surendran et al [35], 2021	N/A	Frequency of: Application use Access to educational lessons Coaching massages received	N/A	Only means, SDs, and percentage results
Carral, et al [30], 2015	Glycemic control: Change in HbA1c Need for insulin Maternal outcomes: Delivery mode—CS, vaginal, or vacuum PIH or pre-eclampsia Gestational week of delivery Weight gain BP	Number of patient visits: GDU^o^ Obstetrics service Emergency General practitioner	Birth weight or macrosomia Neonatal hypoglycemia Preterm birth	Women in the IG required insulin therapy less frequently than women in the CG (P=.02) Women in the IG had a lower number of visits to the GDU (P<.001), nurse educator (P<.001), and general practitioner (P<.001) than patients in the CG
Guo et al [34], 2019	Glycemic control: Change in HbA1c 2-hour glucose level postpartum OGTT Maternal outcomes: Delivery mode—CS, vaginal, or vacuum Gestational week of delivery Weight gain	Compliance Number of outpatient visits	Birth weight or macrosomia Neonatal hypoglycemia Shoulder dystocia	Patient compliance was higher in the IG than in the CG (P<.001) Frequency of outpatient service visits was lower in the IG compared with the CG (P<.001) Weight gain in the IG was lower than in the CG (P<.001) FBG (P<.001) and 2-hour postprandial (P<.001) were lower in the IG than in the CG
Mackillop et al [40], 2018	Glycemic control: Change in HbA1c Longitudinal glycemic control Maternal outcomes: Delivery mode—CS, vaginal, or vacuum PIH or pre-eclampsia Weight gain Gestational week of delivery	Treatment satisfaction Compliance	Birth weight or macrosomia Admission to the NICU Neonatal jaundice or hyperbilirubinemia Neonatal hypoglycemia Shoulder dystocia	Cesarean delivery was lower in the IG compared with the CG (P=.005), with notably fewer emergency cesarean deliveries in the IG Women in the IG had higher satisfaction with care (P=.049) Compliance with BG readings was better in the IG (OR^p^ 2.44, 95% CI 1.29-4.61)
Miremberg et al [41], 2018	Glycemic control: Longitudinal glycemic control Need for insulin Maternal outcomes: Delivery mode—CS, vaginal, or vacuum PIH or pre-eclampsia Gestational week of delivery Polyhydramnios Perinatal tears	Compliance (the actual BG measurements vs instructed measurements)	Birth weight or macrosomia Admission to the NICU Neonatal jaundice or hyperbilirubinemia Neonatal hypoglycemia Shoulder dystocia Neonatal respiratory morbidity Neonatal death	Compliance was higher in the IG than in the CG (P<.001) Mean BG was lower in the IG than in the CG (P<.001) Overall rate of insulin treatment was lower in the IG than in the CG (P=.04) FBG (P<.001) and 1-hour postprandial (P<.001) were lower in the IG than in the CG
Yew et al [36], 2021	Glycemic control: Longitudinal glycemic control Need for insulin Maternal outcomes: Delivery mode—CS, vaginal, or vacuum PIH or pre-eclampsia Weight gain Gestational week of delivery Need for insulin	Mental and emotional health outcomes Depression Anxiety Compliance	Birth weight or macrosomia Admission to the NICU Neonatal jaundice or hyperbilirubinemia Neonatal hypoglycemia Apgar score Shoulder dystocia Neonatal respiratory morbidity Neonatal death	Glucose above the targets was significantly lower in the IG than in the CG (before meal: P=.003; 2 hours after meal: P=.001) Overall, neonatal complications were lower in the IG (38.1%) than in the CG (53.7%; P=.006)
Rasekaba et al [38], 2018	Glycemic control: Longitudinal glycemic control Insulin dose Maternal outcomes: Delivery mode—CS, vaginal, or vacuum	Health service use	Admission to the NICU Macrosomia or infant or birth weight	Women in the CG reached optimal glycemic control (maximum insulin dose) quicker than women in the IG (mean 4.3, SD 4.2 weeks vs mean 7.6, SD 4.5 weeks; P<.001) and had fewer insulin titrations (P=.04)

^a^BG: blood glucose.

^b^FBG: fasting BG.

^c^2hBG: 2-hour BG.

^d^PROM: premature rupture of membranes.

^e^CS: cesarean section.

^f^N/A: not applicable.

^g^IG: intervention group.

^h^CG: control group.

^i^1hBG: 1-hour BG.

^j^PIH: pregnancy-induced hypertension.

^k^NICU: neonatal intensive care unit.

^l^IVR: interactive voice response.

^m^OGTT: oral glucose tolerance test.

^n^BP: blood pressure.

^o^GDU: gestational diabetes unit.

^p^OR: odds ratio.

#### Glycemic Control

Interventions targeting pregnant women with GDM primarily focused on glycemic control. The apps and telemedicine interventions enabled the women to transmit their blood glucose measurements and receive feedback or alerts on their glucose values as well as treatment recommendations. A total of 79% (11/14) of the studies examined glycemic control following the intervention. In total, 14% (2/14) of the studies (WeChat and Guo et al [34]—Dnurse app) reported significant differences in fasting plasma glucose, 2-hour fasting blood glucose, 1-hour postprandial [33,34], and HbA1c before delivery [34]. In 14% (2/14) of the studies (Miremberg et al [41] and Yew et al [36]—Habits-GDM), pregnant women’s longitudinal mean blood glucose values were lower [36,41], and 21% (3/14) of the studies (Carral et al [30]—DiabeTIC, Rasekaba et al [38]—TeleGDM, and Miremberg et al [41]) reported a decrease in the need for insulin therapy in the intervention group.

#### Maternal Outcomes

A total of 79% (11/14) of the studies compared maternal outcomes between women who participated in the intervention and those in the control group receiving usual standard care. The delivery mode included CS, vaginal, or vacuum. Only 9% (1/11) of the studies reported significantly lower rates of cesarean delivery in the intervention group than in the control group (Mackillop et al [40]). Guo et al [34] found significant differences in weight gain in favor of the intervention group in comparison with the control group (Dnurse app).

#### Maternal Lifestyle Measurements

A total of 71% (10/14) of the studies evaluated women’s lifestyle outcomes. These outcomes included engagement with their health condition, depression and anxiety, satisfaction, compliance, use of the system, and changes in the number of clinical visits. In the Pregnant+ app intervention, 84% of the women reported that the app increased engagement with their health compared with 64% in the control group (P<.01) [25]. Another study showed that women in the intervention group transmitted more glucose measurements [37]. A lower number of patient visits was reported in 30% (3/10) of these studies (Pérez-Ferre et al [29], Carral et al [30]—DiabeTIC, and Guo et al [34]—Dnurse app), and higher patient compliance following the intervention was found in 30% (3/10) of the studies (Guo et al [34]—Dnurse app**,** Mackillop et al [40], and Miremberg et al [41]). Overall, satisfaction with care was found to be significantly higher in only 10% (1/10) of the studies (Mackillop et al [40]).

#### Neonatal Outcomes

Neonatal outcomes were examined in 79% (11/14) of the studies. Only 9% (1/11) of these studies found a significantly lower difference in the composite overall neonatal complications (birth trauma, hypoglycemia, hyperbilirubinemia, respiratory distress, neonatal intensive care unit admission, and perinatal death) in the intervention group compared with the control group. However, no differences were found in each outcome individually (Yew et al [36]—Habits-GDM).

## Discussion

### Principal Findings

The use of mHealth apps and digital platforms as a resource for information and pregnancy follow-up among pregnant women is rising. However, digital mHealth interventions require consideration of users’ cognitive and technical skills, education level, and digital literacy level and of cultural appropriateness in addressing dietary preferences and religious customs. These factors are crucial not only to enhance the use of an mHealth app but also to promote better outcomes for pregnant women from diverse ethnic and socioeconomically disadvantaged populations.

This review highlighted that, although existing digital technologies may improve glycemic control and diabetes self-management, there is a consistent deficit in assessing for cultural and digital health literacy appropriateness for pregnant women diagnosed with GDM. Only 17% (2/12) of the interventions addressed language diversity, dietary habits, and culturally appropriate recipes for their patients [36,43]. Only 25% (3/12) of the interventions received a score of ≥3 for digital health literacy appropriateness in our assessment [25,26,30,37]. Owing to the limited evidence that exists, it is hard to understand how participants’ cultural customs and preferences, as well as their level of digital health literacy, affected the interventions’ effectiveness or were associated with better maternal and infant health outcomes.

Assessment of digital health literacy and cultural needs is essential to identify obstacles and barriers to the adoption and use of mHealth and telemedicine systems and enhance the usability of any technology for health care. We found, for example, that only 17% (2/12) of the interventions trained patients on how to use the applications [33,37], and 6% (1/17) of the studies identified technological literacy as a barrier to using the service [50]. In 2021, the World Health Organization released its Global Strategy on Digital Health 2020-2025. The report emphasizes that, as digital health systems and interventions become more common, literacy will become a crucial determinant in the adoption of these technologies [54].

Many different measurements for digital and health literacy exist in the literature [55], but guidelines for the design and development of appropriate digital health interventions, especially for an audience with low literacy levels, are still scarce. In 2001, the US Department of Health and Human Services, Office of Minority Health, published the National Standards for Culturally and Linguistically Appropriate Services in Health and Health Care to assist health care providers in becoming culturally competent and sensitive [56]. These 14 standards are a call to action and include, for example, language assistance services both verbally and in writing as well as training of staff. Thus, although there is increasing awareness of providing suitable care for patients with diverse values, beliefs, and behaviors, it is not yet part of the strategic planning included in the design of digital apps and technologies for women diagnosed with GDM.

It is important to also address the major limitation in GDM studies because of the lack of international consensus between countries (eg, the United States, Europe, and Australia) or health organizations (eg, the American Diabetes Association, American College of Obstetricians and Gynecologists, World Health Organization, European Board, and College of Obstetrics and Gynecology) on GDM screening methods and diagnosis threshold criteria. Different approaches exist, and there is no one standard treatment protocol [57,58]. Therefore, it is difficult to compare the effectiveness of therapies and mHealth and telehealth interventions between different countries because of the variety in the standards of care and the influence of local practices. Tsakirdis et al [59], in their review on national and international guidelines for diagnosis and management of GDM, presented similarities and differences between countries and organizations. In their conclusions, they emphasized the need for future research to resolve guideline conflicts and provide 1 international standard protocol for the screening and management of women with GDM.

An additional limitation we observed in studies reporting on GDM mHealth apps relates to the study sample size. The studies included in this review did not report in the methods section on the power analysis calculation that they conducted to determine their study sample size. Moreover, many studies (5/13, 38%) reported on small intervention groups. A small sample size can result in an underpowered study, which can lead to biased conclusions regarding the effectiveness of the interventions.

### Strengths and Limitations

This review has several strengths and limitations that need to be acknowledged. The framework we used provided us with the foundation for a rigorous and transparent method to conduct this scoping review. A comprehensive literature search was conducted, and the papers retrieved were screened and selected by 2 independent reviewers (YS and SS). Both reviewers met on a regular basis for discussions and to resolve disagreements. However, our search was restricted to the English language. Manuscripts that were published in other languages were excluded, and it could be that their interventions were relevant. An additional limitation may be a result of our search focusing on studies published between 2010 and 2021. Technologies evolve and change day by day. Computers and mobile phone generations continue to improve over time, and there may be a difference in our evaluation because of improved technological abilities over the years.

### Conclusions and Future Directions

This review explored the published literature on digital interventions for GDM. Although studies on digital technologies for health self-management exist, this review found only 12 published interventions and fewer studies that evaluated and designed the technology for pregnant women diagnosed with GDM in accordance with the patients’ cultural needs and digital health literacy levels. Thus, there is insufficient evidence regarding the effectiveness and benefits of mHealth and telemedicine systems for women from diverse backgrounds. Future research is needed to better understand how best to adapt and implement cultural and literacy factors in the design of digital technology for GDM management.

## Appendix

Multimedia Appendix 1 PRISMA (Preferred Reporting Items for Systematic Reviews and Meta-Analyses) extension for Scoping Reviews (PRISMA-ScR): checklist and explanation.

Multimedia Appendix 2 Online search strategy conducted in October 2021.

## Abbreviations

GDM: gestational diabetes mellitus
mHealth: mobile health
PRISMA: Preferred Reporting Items for Systematic Reviews and Meta-Analyses
PRISMA-ScR: Preferred Reporting Items for Systematic Reviews and Meta-Analyses extension for Scoping Reviews
RCT: randomized controlled trial
