# Supplementary material for: Cultural and Digital Health Literacy Appropriateness of App- and Web-Based Systems Designed for Pregnant Women With Gestational Diabetes Mellitus: Scoping Review
Source: J Med Internet Res. 2022 Oct 14;24(10):e37844. doi: 10.2196/37844 (PMC9617190; doi:10.2196/37844)
Supplement: Multimedia Appendix 2 [file jmir_v24i10e37844_app2.docx]

Pubmed online search strategy conducted on October 2021

| Keywords | # |
| --- | --- |
| "Telemedicine" [Mesh] OR "Mobile Applications" [Mesh] OR "mobile application*" [tw] OR "mobile app" [tw] OR "mobile apps" [tw] OR "mobile technolog*" [tw] OR "mobile healthcare" [tw] OR "mHealth" [tw] | 1 |
| "Pregnancy in Diabetics"[Mesh] OR "Diabetes, Gestational"[Mesh] OR "Gestational  diabetes mellitus" [tw] OR "Gestational diabe*" [tw] OR "Diabetes, Pregnancy  Induced" [tw] OR "Diabetes, Pregnancy-Induced” [tw] | 2 |
| "Health Literacy"[Mesh] OR "Digital literacy" [tw] OR "Health Liter*"[tw] OR "health  knowledge" [tw] OR "health understanding" [tw] | 3 |
| #1 AND #2 AND #3 | 4 |
| "Culture" [Mesh] OR "Cultural diversity" [tw] OR “Cultural deprivation" [tw] OR "Cultural competency" [tw] OR "Cultural characte*" [tw] OR "cultural sensitivity" [tw] OR "cultural concordance" [tw] | 5 |
| #1 AND #2 AND #5 | 6 |
| "Vulnerable Populations"[Mesh] OR "Underserved populations" [tw] OR "Underserved Patient" [tw] OR "Disadvantage population" [tw] OR "minority" [tw] OR "minorities" [tw] "race" [tw] OR "ethnicity" [tw] OR "Hispanic Americans"[Mesh] OR "American, Hispanic" [tw] OR "Spanish Americans" [tw] OR "Latin*" [tw] OR "Hispanic" [tw] "Arabs"[Mesh] OR "Arab*" [tw] OR "Palestinian" [tw] OR "Bedouin" [tw] OR "Asian Americans" [Mesh] OR "Americans, Asian" [tw] OR "Religion" [Mesh] OR "Religious Beliefs" [tw] | 7 |
| #1 AND #2 AND #7 | 8 |
| "Healthcare Disparities"[Mesh] OR "Health Care Inequality" [tw] OR "Healthcare Inequality" [tw] OR "Healthcare Disparity" [tw] OR "Health care Disparity" [tw] OR "Health inequity" [tw] | 9 |
| #1 AND #2 AND #9 | 10 |
| "Rural Population"[Mesh] OR "Rural Spatial Distribution" [tw] OR "Rural Community" [tw] OR "Rural Communities" [tw] OR "Poverty Areas" OR "Periphery" [tw] | 11 |
| #1 AND #2 AND #11 | 12 |
| Filters: English, from 2010/1/1 – 2022/1/31 | * |

Medline online search strategy conducted on October 2021

| Keywords | # |
| --- | --- |
| telemedicine or telehealth or ehealth or e-health or mhealth or m-health | S1 |
| gestational diabetes or gdm or gestational diabetes mellitus or diabetes in pregnancy | S2 |
| S1 AND S2 | S3 |
| culture or cultural or ethnicity or identity or values or language or religious or belief | S4 |
| S1 AND S2 AND S4 | S5 |
| health literacy or health education or health knowledge or health information or health understanding or education or educational group | S6 |
| S1 AND S2 AND S6 | S7 |
| digital literacy or computer literacy or technological literacy or internet literacy or digital inclusion | S8 |
| S1 AND S2 AND S8 | S9 |
| cultural diversity in healthcare | S10 |
| S1 AND S2 AND S10 | S11 |
| cultural competence or cultural awareness or cultural competency or cultural sensitivity | S12 |
| S1 AND S2 AND S12 | S13 |
| Vulnerable population | S14 |
| S1 AND S2 AND S14 | S15 |
| underserved populations or minorities or race or ethnicity | S16 |
| S1 AND S2 AND S16 | S17 |
| healthcare disparities or health disparities or poor or poverty or socioeconomic | S18 |
| S1 AND S2 AND S1 | S19 |

Scopus online search strategy conducted on October 2021

| Keywords | # |
| --- | --- |
| TITLE-ABS-KEY ( "telemedicine" OR "mobile application*" OR "mobile app" OR "mobile apps" OR "mobile technolog*" OR "mobile healthcare" OR mhealth "mobile applications" OR "mobile application*" OR "mobile app" OR "mobile apps" OR "mobile technolog*" OR "mobile healthcare" OR mhealth ) | 1 |
| TITLE-ABS-KEY ( "pregnancy in diabetics" OR "diabetes, gestational" OR "gestational diabetes mellitus" OR "gestational diabe*" OR "diabetes, pregnancy induced" OR "diabetes, pregnancy-induced" ) | 2 |
| TITLE-ABS-KEY ( "health literacy" OR "digital literacy" OR "health liter*" OR "health knowledge" OR "health understanding" ) | 3 |
| ( TITLE-ABS-KEY ( "telemedicine" OR "mobile application*" OR "mobile app" OR "mobile apps" OR "mobile technolog*" OR "mobile healthcare" OR mhealth "mobile applications" OR "mobile application*" OR "mobile app" OR "mobile apps" OR "mobile technolog*" OR "mobile healthcare" OR mhealth ) ) AND ( TITLE-ABS-KEY ( "pregnancy in diabetics" OR "diabetes, gestational" OR "gestational diabetes mellitus" OR "gestational diabe*" OR "diabetes, pregnancy induced" OR "diabetes, pregnancy-induced" ) ) AND ( TITLE-ABS-KEY ( "health literacy" OR "digital literacy" OR "health liter*" OR "health knowledge" OR "health understanding" ) ) | 4 |
| TITLE-ABS-KEY ( "culture" OR "cultural diversity" OR "cultural deprivation" OR "cultural competency" OR "cultural characte*" OR "cultural sensitivity" OR "cultural concordance" ) | 5 |
| ( TITLE-ABS-KEY ( "telemedicine" OR "mobile application*" OR "mobile app" OR "mobile apps" OR "mobile technolog*" OR "mobile healthcare" OR mhealth "mobile applications" OR "mobile application*" OR "mobile app" OR "mobile apps" OR "mobile technolog*" OR "mobile healthcare" OR mhealth ) ) AND ( TITLE-ABS-KEY ( "pregnancy in diabetics" OR "diabetes, gestational" OR "gestational diabetes mellitus" OR "gestational diabe*" OR "diabetes, pregnancy induced" OR "diabetes, pregnancy-induced" ) ) AND ( TITLE-ABS-KEY ( "culture" OR "cultural diversity" OR "cultural deprivation" OR "cultural competency" OR "cultural characte*" OR "cultural sensitivity" OR "cultural concordance" ) ) | 6 |
| TITLE-ABS-KEY (vulnerable AND populations OR underserved AND populations OR underserved AND patient OR disadvantage AND population OR minority OR minorities OR race OR ethnicity )KEY ( vulnerable AND populations OR underserved AND populations OR underserved AND patient OR disadvantage AND population OR minority OR minorities OR race OR ethnicity ) | 7 |
| TITLE-ABS-KEY ( "telemedicine" OR "mobile application*" OR "mobile app" OR "mobile apps" OR "mobile technolog*" OR "mobile healthcare" OR mhealth "mobile applications" OR "mobile application*" OR "mobile app" OR "mobile apps" OR "mobile technolog*" OR "mobile healthcare" OR mhealth ) ) AND ( TITLE-ABS-KEY ( "pregnancy in diabetics" OR "diabetes, gestational" OR "gestational diabetes mellitus" OR "gestational diabe*" OR "diabetes, pregnancy induced" OR "diabetes, pregnancy-induced" ) ) AND ( TITLE-ABS-KEY ( vulnerable AND populations OR underserved AND populations OR underserved AND patient OR disadvantage AND population OR minority OR minorities OR race OR ethnicity ) ) | 8 |
| TITLE-ABS-KEY ( "healthcare disparities" OR "health care inequality" OR "healthcare inequality" OR "healthcare disparity" OR "health care disparity" OR "health care inequity" OR "healthcare inequity" ) | 9 |
| ( TITLE-ABS-KEY ( "telemedicine" OR "mobile application*" OR "mobile app" OR "mobile apps" OR "mobile technolog*" OR "mobile healthcare" OR mhealth "mobile applications" OR "mobile application*" OR "mobile app" OR "mobile apps" OR "mobile technolog*" OR "mobile healthcare" OR mhealth ) ) AND ( TITLE-ABS-KEY ( "pregnancy in diabetics" OR "diabetes, gestational" OR "gestational diabetes mellitus" OR "gestational diabe*" OR "diabetes, pregnancy induced" OR "diabetes, pregnancy-  induced" ) ) AND ( TITLE-ABS-KEY ( "healthcare disparities" OR "health care inequality" OR "healthcare inequality" OR "healthcare disparity" OR "health care disparity" OR "health care inequity" OR "healthcare inequity" ) ) | 10 |
| TITLE-ABS-KEY ( "socioeconomic factors" OR "social inequality" | 11 |
| ( TITLE-ABS-KEY ( "telemedicine" OR "mobile application*" OR "mobile app" OR "mobile apps" OR "mobile technolog*" OR "mobile healthcare" OR mhealth "mobile applications" OR "mobile application*" OR "mobile app" OR "mobile apps" OR "mobile technolog*" OR "mobile healthcare" OR mhealth ) ) AND ( TITLE-ABS-KEY ( "pregnancy in diabetics" OR "diabetes, gestational" OR "gestational diabetes mellitus" OR "gestational diabe*" OR "diabetes, pregnancy induced" OR "diabetes, pregnancy-induced" ) ) AND ( TITLE-ABS-KEY ( "socioeconomic factors" OR "social inequality" ) ) | 12 |
| TITLE-ABS-KEY ( "rural population" OR "rural spatial distribution" OR "rural community" OR "rural communities" OR "periphery" OR "social periphery" ) | 13 |
| ( TITLE-ABS-KEY ( "telemedicine" OR "mobile application*" OR "mobile app" OR "mobile apps" OR "mobile technolog*" OR "mobile healthcare" OR mhealth "mobile applications" OR "mobile application*" OR "mobile app" OR "mobile apps" OR "mobile technolog*" OR "mobile healthcare" OR mhealth ) ) AND ( TITLE-ABS-KEY ( "pregnancy in diabetics" OR "diabetes, gestational" OR "gestational diabetes mellitus" OR "gestational diabe*" OR "diabetes, pregnancy induced" OR "diabetes, pregnancy-induced" OR "gdm" ) ) AND ( TITLE-ABS-KEY ( "rural population" OR "rural spatial distribution" OR "rural community" OR "rural communities" OR "periphery" OR "social periphery" ) ) | 14 |
| "poverty areas" | 15 |
| ( TITLE-ABS-KEY ( "telemedicine" OR "mobile application*" OR "mobile app" OR "mobile apps" OR "mobile technolog*" OR "mobile healthcare" OR mhealth "mobile applications" OR "mobile application*" OR "mobile app" OR "mobile apps" OR "mobile technolog*" OR "mobile healthcare" OR mhealth ) ) AND ( TITLE-ABS-KEY ( "pregnancy in diabetics" OR "diabetes, gestational" OR "gestational diabetes mellitus" OR "gestational diabe*" OR "diabetes, pregnancy induced" OR "diabetes, pregnancy-induced" OR "gdm" ) ) AND ( TITLE-ABS-KEY ( "poverty areas" ) ) | 16 |
| TITLE-ABS-KEY ( "hispanic americans" OR "american, hispanic" OR "spanish americans" OR "latino" OR "latin*" OR "latina" OR "hispanic" ) | 17 |
| ( TITLE-ABS-KEY ( "telemedicine" OR "mobile application*" OR "mobile app" OR "mobile apps" OR "mobile technolog*" OR "mobile healthcare" OR mhealth "mobile applications" OR "mobile application*" OR "mobile app" OR "mobile apps" OR "mobile technolog*" OR "mobile healthcare" OR mhealth ) ) AND ( TITLE-ABS-KEY ( "pregnancy in diabetics" OR "diabetes, gestational" OR "gestational diabetes mellitus" OR "gestational diabe*" OR "diabetes, pregnancy induced" OR "diabetes, pregnancy-induced" OR "gdm" ) ) AND ( TITLE-ABS-KEY ( "hispanic americans" OR "american, hispanic" OR "spanish americans" OR "latino" OR "latin*" OR "latina" OR "hispanic" ) ) | 18 |
| TITLE-ABS-KEY ( "asian americans" OR "americans, asian" ) | 19 |
| ( TITLE-ABS-KEY ( "telemedicine" OR "mobile application*" OR "mobile app" OR "mobile apps" OR "mobile technolog*" OR "mobile healthcare" OR mhealth "mobile applications" OR "mobile application*" OR "mobile app" OR "mobile apps" OR "mobile technolog*" OR "mobile healthcare" OR mhealth ) ) AND ( TITLE-ABS-KEY ( "pregnancy in diabetics" OR "diabetes, gestational" OR "gestational diabetes mellitus" OR "gestational diabe*" OR "diabetes, pregnancy induced" OR "diabetes, pregnancy-induced" OR "gdm" ) ) AND ( TITLE-ABS-KEY ( "asian americans" OR "americans, asian" ) ) | 20 |
|  |  |
| TITLE-ABS-KEY ( "religion" OR "religious beliefs" ) | 21 |
| ( TITLE-ABS-KEY ( "telemedicine" OR "mobile application*" OR "mobile app" OR "mobile apps" OR "mobile technolog*" OR "mobile healthcare" OR mhealth "mobile applications" OR "mobile application*" OR "mobile app" OR "mobile apps" OR "mobile technolog*" OR "mobile healthcare" OR mhealth ) ) AND ( TITLE-ABS-KEY ( "pregnancy in diabetics" OR "diabetes, gestational" OR "gestational diabetes mellitus" OR "gestational diabe*" OR "diabetes, pregnancy induced" OR "diabetes, pregnancy-induced" OR "gdm" ) ) AND ( TITLE-ABS-KEY ( "religion" OR "religious beliefs" ) ) | 22 |
| TITLE-ABS-KEY ( "arabs" OR "arab" OR "palestinian" OR "bedouin" ) | 23 |
| TITLE-ABS-KEY ( "telemedicine" OR "mobile application*" OR "mobile app" OR "mobile apps" OR "mobile technolog*" OR "mobile healthcare" OR mhealth "mobile applications" OR "mobile application*" OR "mobile app" OR "mobile apps" OR "mobile technolog*" OR "mobile healthcare" OR mhealth ) ) AND ( TITLE-ABS-KEY ( "pregnancy in diabetics" OR "diabetes, gestational" OR "gestational diabetes mellitus" OR "gestational diabe*" OR "diabetes, pregnancy induced" OR "diabetes, pregnancy-induced" OR "gdm" ) ) AND ( TITLE-ABS-KEY ( "arabs" OR "arab" OR "palestinian" OR "bedouin" ) ) | 24 |
| TITLE-ABS-KEY ( "emigrants and immigrants" OR "immigrants" OR "foreigner" OR "alien" OR "emigrant" ) | 25 |
| ( TITLE-ABS-KEY ( "telemedicine" OR "mobile application*" OR "mobile app" OR "mobile apps" OR "mobile technolog*" OR "mobile healthcare" OR mhealth "mobile applications" OR "mobile application*" OR "mobile app" OR "mobile apps" OR "mobile technolog*" OR "mobile healthcare" OR mhealth ) ) AND ( TITLE-ABS-KEY ( "pregnancy in diabetics" OR "diabetes, gestational" OR "gestational diabetes mellitus" OR "gestational diabe*" OR "diabetes, pregnancy induced" OR "diabetes, pregnancy-induced" OR "gdm" ) ) AND ( TITLE-ABS-KEY ( "emigrants and immigrants" OR "immigrants" OR "foreigner" OR "alien" OR "emigrant" ) ) | 26 |
| TITLE-ABS-KEY ( "african americans" OR "ethnic groups" ) | 27 |
| ( TITLE-ABS-KEY ( "telemedicine" OR "mobile application*" OR "mobile app" OR "mobile apps" OR "mobile technolog*" OR "mobile healthcare" OR mhealth "mobile applications" OR "mobile application*" OR "mobile app" OR "mobile apps" OR "mobile technolog*" OR "mobile healthcare" OR mhealth ) ) AND ( TITLE-ABS-KEY ( "pregnancy in diabetics" OR "diabetes, gestational" OR "gestational diabetes mellitus" OR "gestational diabe*" OR "diabetes, pregnancy induced" OR "diabetes, pregnancy-induced" OR "gdm" ) ) AND ( TITLE-ABS-KEY ( "african americans" OR "ethnic groups" ) ) | 28 |

Web of science online search strategy conducted on October 2021

| Keywords | # |
| --- | --- |
| **"Telemedicine" OR "mobile application*" OR "mobile app" OR "mobile apps" OR "mobile technolog*" OR "mobile healthcare" OR mHealth** (Topic) | 1 |
| **"Telemedicine" OR "mobile application*" OR "mobile app" OR "mobile apps" OR "mobile technolog*" OR "mobile healthcare" OR mHealth** (Topic) and **English** (Languages) | 2 |
| **"Telemedicine" OR "mobile application*" OR "mobile app" OR "mobile apps" OR "mobile technolog*" OR "mobile healthcare" OR mHealth** (Topic) and **English** (Languages) and **2022** or **2021** or **2020** or **2019** or **2018** or **2017** or **2016** or **2015** or **2014** or **2013** or **2012** or **2011** or **2010** (Publication Years) | 3 |
| **TS=("Pregnancy in Diabetics" OR "Diabetes, Gestational" OR "Gestational diabetes mellitus" OR "Gestational diabe*" OR "Diabetes, Pregnancy Induced" OR "Diabetes, Pregnancy-Induced" )** | 4 |
| **TS=(Culture OR Cultural diversity OR Cultural deprivation OR Cultural competency OR Cultural characteristic OR cultural sensitivity OR cultural concordance)** | 5 |
| **#3 AND #4 AND #5** | 6 |
| TS=("Health Literacy" OR "Digital literacy" OR "Health Liter*" OR "health knowledge" OR "health understanding") | 7 |
| #3 AND #4 AND #7 | 8 |
| TS=("Vulnerable Populations" OR "Underserved populations" OR "Underserved Patient" OR "Disadvantage population" OR "minority" OR "minorities" "race" OR "ethnicity" ) | 9 |
| #3 AND #4 AND #9 | 10 |
| TS=(Healthcare Disparities OR Health Care Inequality OR Healthcare Inequality OR Healthcare Disparity OR Health care Disparity OR health care inequity OR healthcare inequity | 11 |
| #3 AND #4 AND #11 | 12 |
| TS=(Socioeconomic Factors) | 13 |
| #3 AND #4 AND #13 | 14 |
| TS=("Rural Population" OR "Rural Spatial Distribution" OR "Rural Community" OR "Rural Communities" OR "periphery" OR "Social periphery") | 15 |
| #3 AND #4 AND #15 | 16 |
| TS=(Poverty Areas) | 17 |
| #3 AND #4 AND #17 | 18 |
| TS=("Hispanic Americans" OR "American, Hispanic" OR "Spanish Americans" OR "Latino" OR "Latin*" OR "Latina" OR "Hispanic" ) | 19 |
| #3 AND #4 AND #19 | 20 |
| TS=("Asian Americans" OR "Americans, Asian" ) | 21 |
| #3 AND #4 AND #21 | 22 |
| TS=("Religion" OR "Religious Beliefs" ) | 23 |
| #3 AND #4 AND #23 | 24 |
| TS=("Arabs" OR "Arab" OR "Palestinian" OR "Bedouin" ) | 25 |
| #3 AND #4 AND #25 | 26 |
| TS=("Emigrants and Immigrants" OR "Immigrants" OR "Foreigner" OR "Alien" OR "Emigrant" ) | 27 |
| #3 AND #4 AND #27 | 28 |
| TS=("African Americans" OR "Ethnic Groups") | 29 |
| #3 AND #4 AND #29 | 30 |
